# The tetrameric peptide LfcinB (20–25)_4_ derived from bovine lactoferricin induces apoptosis in the MCF-7 breast cancer cell line

**DOI:** 10.1039/c9ra04145a

**Published:** 2019-07-01

**Authors:** Jorge Rodríguez Guerra, Andrea Barragán Cárdenas, Alejandra Ochoa-Zarzosa, Joel López Meza, Adriana Umaña Pérez, Ricardo Fierro-Medina, Zuly Jenny Rivera Monroy, Javier Eduardo García Castañeda

**Affiliations:** Pharmacy Department, Universidad Nacional de Colombia Carrera 45 No. 26-85, Building 450, Office 213 11321 Bogotá Colombia jaegarciaca@unal.edu.co +57-1-316-5000 ext. 14436; Chemistry Department, Universidad Nacional de Colombia Carrera 45 No. 26-85, Building 451, Office 409 11321 Bogotá Colombia; Multidisciplinary Center for Studies in Biotechnology, Faculty of Veterinary Medicine and Zootechnics, Universidad Michoacana de San Nicolás de Hidalgo Km 9.5 Carretera Morelia-Zinapécuaro Mexico

## Abstract

The cytotoxic effect of the tetrameric peptide LfcinB (20–25)_4_ against breast cancer cell line ATCC® HTB-22™ (MCF-7) was evaluated. The tetrameric peptide exhibited a concentration-dependent cytotoxic effect against MCF-7 cancer cells. The peptide at 22 µM had the maximum cytotoxic effect against MCF-7 cancer cells, reducing their cell viability to ∼20%. The cytotoxic effect of the tetrameric peptide against MCF-7 cells was sustained for 24 hours. Furthermore, the tetrameric peptide did not exhibit a significant cytotoxic effect against the non-tumorogenic trophoblastic cell line, which confirms their selectivity for breast cancer cell lines. The MCF-7 cells treated at 12.2 µM for 1 h exhibited morphological changes characteristic of apoptosis, such as rounded forms and cellular shrinkage. Furthermore, this peptide induces severe cellular damage to MCF-7 cells, mitochondrial membrane depolarization, and increase of cytoplasmic calcium concentration. Our results suggest that it has a significant selective cytotoxic effect against MCF-7 cells, which may be mainly associated with the apoptotic pathway. This peptide, which contains the RRWQWR motif, could be considered to be a promising candidate for developing therapeutic agents for the treatment of breast cancer.

## Abstract 2

The cytotoxic effect of the tetrameric peptide LfcinB (20–25)_4_ against breast cancer cell line ATCC® HTB-22™ (MCF-7) was evaluated.

## Graphical abstract

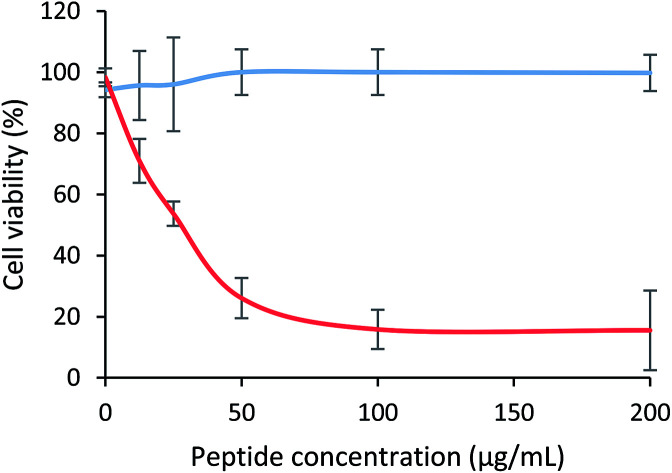

## Introduction

According to the World Health Organization (WHO), breast cancer is the second most frequent cancer, impacting over 2 million people each year. Breast cancer is the most common cause of death in women; about 600 000 women died from it in 2018.^1^ Current treatments include surgery, chemotherapy, radiotherapy, and hormone therapy, which are combined according to the patient. These therapies have increased the survival rate and reduced recurrence. However, breast cancer incidence is continuously increasing and is a serious public health problem. The treatments produce severe side effects, significantly affecting the patients' quality of life.

Surgery and radiotherapy are invasive procedures that cause several psychological and physical effects such as pain, fatigue, and inflammation,^2^ and the same is true for chemotherapy patients, who can suffer neutropenia, nausea and vomiting, amenorrhea, alopecia, neurological toxicity, weight gain, secondary leukemia, and cardiotoxicity.^3,4^ Hormone therapy is effective only in those cancers with hormone receptors and causes hot flashes, musculoskeletal pain, fatigue, mood disturbances, nausea, vomiting, and fractures, among other complications.^5^

Due to the foregoing, it is imperative to find new strategies for developing selective and effective drugs that do not significantly compromise the patient's quality of life. Antimicrobial peptides (AMPs) are a promising alternative, due to their ability to establish different points of contact with their target and selective interactions that will generate a decrease in side effects.^6^ AMPs are part of the innate immune system and exhibit a cytotoxic effect against bacteria and cancer cells, and are a viable alternative for identifying promising molecules for developing new therapeutic agents.^7,8^ AMPs are able to discriminate between cancer and non-cancer cells due to electrostatic interactions between positively-charged amino acids and the negatively-charged membrane components such as phosphatidylserine, glycosylated mucins, sialylated gangliosides, sialic acid, and heparan sulphate.^9,10^

Bovine lactoferricin (LfcinB) ^17^FKCRRWQWRMKKLGAPSITCVRRAF^41^ is an AMP that is a product of bovine lactoferrin (BLF) hydrolysis.^11,12^ It has been reported that LfcinB exhibited higher antimicrobial and anticancer activity than native protein. The action mechanism proposed for LfcinB antibacterial activity suggests that positively-charged residues (Arg) interact with the negatively-charged lipopolysaccharide of the bacterial cell wall, allowing the peptide to approach the bacterial membrane; then hydrophobic residues (Trp) of the peptide interact with the lipid bilayer, causing membrane disruption or instability. The peptide's interaction with the membrane can induce cellular lysis or peptide internalization, suggesting that the peptide's antibacterial activity also may include intracellular targets.^13,14^

LfcinB exhibits a cytotoxic effect against human cell lines derived from breast, gastric, leukaemia, fibrosarcomas, melanomas, and colon cancer and inhibits metastasis in the liver and lungs.^15–20^ Furthermore, LfcinB has not exhibited a cytotoxic effect in primary cultures of lymphocytes, fibroblasts, or human endothelial cells.^16,19,21–24^ The LfcinB cytotoxic effect has been attributed to the minimal motif ^20^RRWQWR^25^, which is an amphipathic and positively-charged sequence.^25–27^ However, previous studies demonstrated that the RRWQWR peptide does not exhibit a cytotoxic effect against either MDA-MB-231 or Jurkat T cells, while intracellular delivery of LcinB (20–25) induced a cytotoxic effect in these cells lines.^25^ Furthermore, the polyvalence of the minimal motif (dimer and tetramer) significantly increased the selective cytotoxic effect against oral and breast cancer cells.^16^

In previous studies, we have reported that the tetrameric peptide LfcinB (20–25)_4_ exhibited antibacterial activity against Gram-positive and Gram-negative bacterial strains.^16^ Furthermore, this peptide exhibited a significant cytotoxic effect against CAL-27 and SCC15 cell lines. The peptide's cytotoxic effect was fast and concentration dependent and was maintained for 24 h, while the cytotoxic effect against non-tumorigenic cell lines HET-1 was moderate. Transmission electron microscopy assays show structural damage caused by this peptide in CAL-27 cells; disruption of the cell membrane and severe damage to the cytoplasm and the nucleus were observed. Cell lysis was evident after 15 min of treatment, suggesting that the cytotoxic effect is fast. In an *in vivo* model, this peptide exhibited antitumoral activity, which was associated with apoptotic and necrotic processes.^28^ This peptide also exhibited a significant cytotoxic effect against MDA-MB-468 (IC_50_ = 6 µM) and MDA-MB-231 (IC_50_ = 15 µM) breast cancer cell lines. It was observed that cell viability was near zero when cells were treated with the tetrameric peptide at 11 µM.^16^ Additionally, this peptide did not exhibit a significant cytotoxic effect against the dermal fibroblast human cell line PCS 201-102, which confirms their selectivity for cancer cells.^16^

In the present paper, the cytotoxic effect of the tetrameric peptide LfcinB (20–25)_4_ against breast cancer cell line ATCC® HTB-22™ (MCF-7) and human trophoblast cell line ATCC® CRL-3271™ was evaluated. In addition, we did a preliminary study of the action mechanism related to the peptide's cytotoxic effect. The results suggest that the tetrameric peptide LfcinB (20–25)_4_ is a promising molecule for developing therapeutic agents against breast cancer.

## Results and discussion

**Fig. 1:**
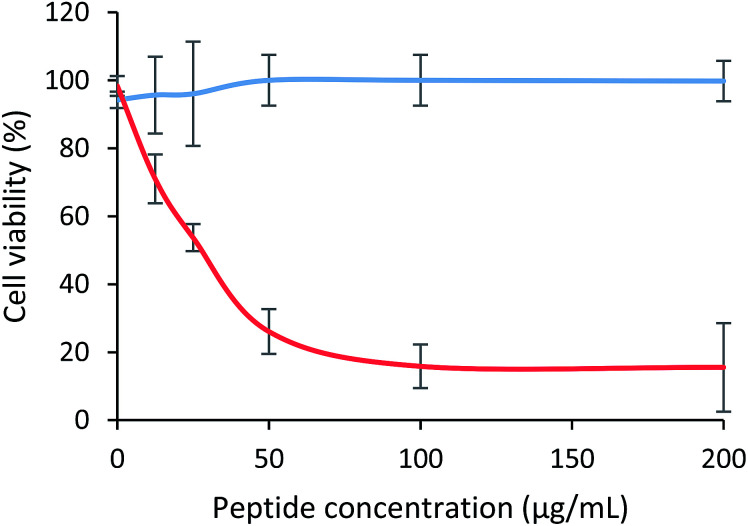
Cytotoxic effect of LfcinB (21–25)_4_ peptide against MCF-7 and CRL-3271 cell lines after 2 h treatment at 37 °C. Cell viability percentage of breast cancer cell line MCF-7 (red) and human trophoblast cell line CRL-3271 (blue) can be observed. The data are expressed as the mean ± SD (*n* = 3). Statistically significant differences were found between the cytotoxic effect exhibited by the tetrameric peptide in both cell lines at 12.5 µg mL^−1^* and 25, 50, 100 and 200 µg mL^−1^**** (ANOVA, Sidak's multiple comparisons test, *p < 0.05; ****p < 0.0001).

The cytotoxic effect of the tetrameric peptide LfcinB (20–25)_4_ against breast cancer cell line MCF-7 and human trophoblast cell line CRL-3271™ was evaluated (Fig. 1). When MCF-7 cells were treated with the peptide (1–44 µM) for 2 h, a significant reduction in cell viability was observed. The peptide's cytotoxic effect was concentration-dependent in the 1–11 µM range, the IC_50_ being 6.5 µM (∼30 µg mL^−1^). The maximum cytotoxic effect (∼20% cell viability) was reached at a peptide concentration between 11 and 44 µM. Contrary to this, in the CRL-3271™ cell line, no significant cytotoxic effect of the peptide was observed, since cell viability was not significantly affected at the peptide concentrations evaluated.

**Fig. 2:**
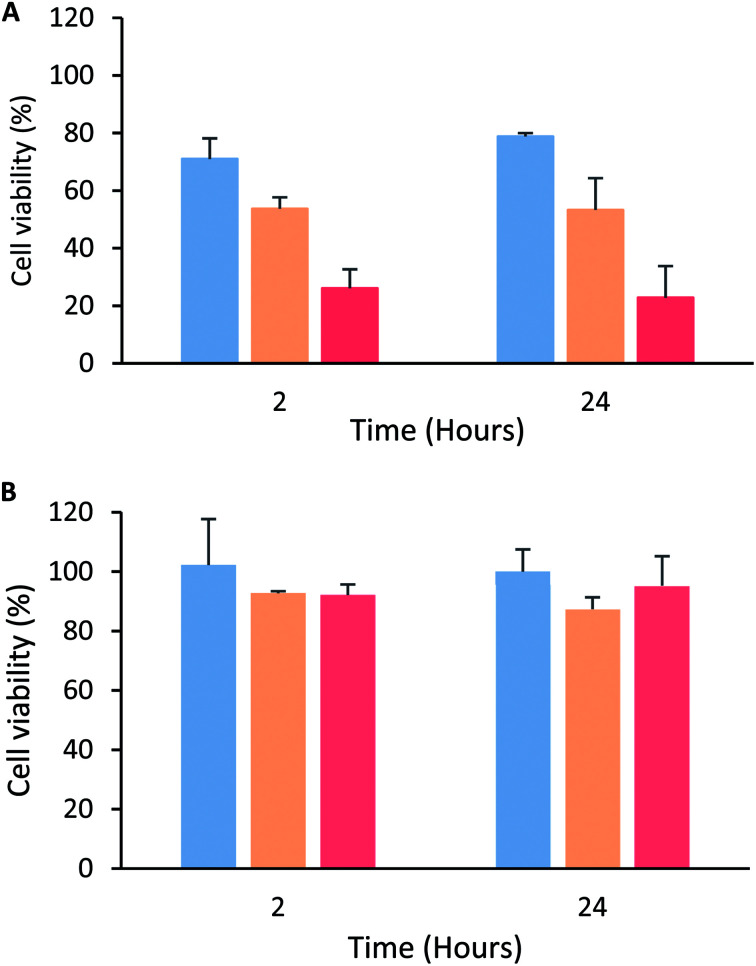
Cytotoxic effect of peptide LfcinB (21–25)_4_ against MCF-7 and CRL-3271 cells after 2 and 24 h treatment. (A) MCF-7 cells were treated with peptide at concentrations: 15 µg mL^−1^/3.3 µM (blue); 30 µg mL^−1^/6.6 µM (orange) and 60 µg mL^−1^/13.3 µM (red). (B) CRL-3271 cells were treated with peptide concentrations: at 5.5 µM (blue), 11 µM (orange) and 22 µM (red). Treatment time ± SD (*n* = 3) ANOVA *post hoc* Tukey, *p* < 0.05 *statistic significant differences with regard to maximum cytotoxic effect.

The peptide's cytotoxic effect against MCF-7 cells by modifying the treatment time (2, 6, 12 and 24 h) and peptide concentration 15 µg mL^−1^ (3.3 µM), 30 µg mL^−1^ (6.6 µM) and 60 µg mL^−1^ (13.3 µM) was evaluated. The cytotoxic effect was concentration-dependent in all cases, and no significant differences in the peptide's cytotoxic effect during the period of time evaluated were observed, suggesting that the peptide maintained its cytotoxic effect for 24 h (Fig. 2A). A similar behavior also was reported by Solarte *et al.*,^29^ the cytotoxic effect of tetrameric peptide against CAL-27 cells was concentration dependent and maintained until 24 h. In the same way, Vargas *et al.*,^16^ reported the time–kill curve of *S. aureus* treated with the tetrameric peptide and it was observed that after 48 h the LfcinB (20–25)_4_ exhibited bactericide effect at 44 µM, and bacteriostatic at 22 µM. These results suggest that the tetramer activity dependents of its concentration and it is maintained during the evaluated time, evidencing that the peptide integrity is not affected in the culture medium during the evaluated time (24–48 h). In order to establish if the tetrameric peptide's cytotoxic effect is selective for MCF-7 cells, the tetrameric peptide's cytotoxic effect against CRL-3271 cells at 24 h treatment was evaluated. When the CRL-3271 cells were treated with the tetrameric peptide, the cell viability percentage was near 80% in all cases, indicating that the peptide's cytotoxic effect against CRL-3271 was lower at both peptide concentrations and treatment times evaluated (Fig. 2B). Thus the LfcinB (20–25)_4_ peptide exhibits a selective cytotoxic effect against the MCF-7 cancer cells without affecting the human trophoblast cell line.

These results are in agreement with those obtained previously by us; the tetrameric peptide exhibited a selective cytotoxic effect against breast cancer cell lines MDA-MB-468 and MDA-MB-231, IC_50_ being 4 and 6 µM, respectively.^16^ Furthermore, the LfcinB (20–25)_4_ peptide doesn't exhibit a significant cytotoxic effect against human dermal fibroblast cells PCS 201-012, suggesting that the tetrameric peptide LfcinB (20–25)_4_ exhibits a selective cytotoxic effect against human cell lines derived from breast cancer.^16^ These results indicate that the peptide exhibited a selective cytotoxic effect against MCF-7, MDA-MB-468, and MDA-MB-231 breast cancer cells, suggesting that it has a broad spectrum. These breast cancer cell lines belong to subtypes related to high incidence in the diagnosed cases; MDA-MB-468 and MDA-MB-231 cells were isolated from an adenocarcinoma and belong to the triple-negative subtype, which has been identified in 10–20% of the cases diagnosed, while the MCF-7 cells are classified as ER+, PR+/−, and HER2-subtypes, which have been identified in 60–80% of cases reported for breast cancer.^30,31^

On the other hand, this peptide also exhibited a selective cytotoxic effect against OSCC-derived cell lines CAL-27 (IC_50_ = 9 µM) and SCC15 (IC_50_ = 9 µM), while the peptide's cytotoxic effect against the non-tumorigenic cell line was moderate.^28,29^ It can be seen that these IC_50_ values are of the same order of magnitude as those obtained for MCF-7 cells, which confirms that the peptide exhibited significantly selective cytotoxic effect against human cell lines derived from breast and oral cancer. The cytotoxic effect of the peptide against MCF-7, CAL-27, and SCC-15 cancer cells continues for 24 h after treatment, suggesting that the peptide's cytotoxic effect against cancer cells lines could be broad spectrum.^28,29^

The tetrameric peptide LfcinB (20–25)_4_ is a tetra-branched molecule containing the minimal motif RRWQWR in each branch. In a similar way, in addition to the tetrameric peptide LfcinB (20–25)_4_, other polyvalent peptides (dimeric and tetrameric peptides) containing RRWQWRMKLG and FKARRWQWRMKKLG sequences, which include the minimal motif, also exhibited a significant selective cytotoxic effect against breast cancer cells MDA-MB-468 and MDA-MB-231, suggesting that the polyvalence of the RRWQWR sequence is relevant for the cytotoxic effect against breast cancer cells.^16^ Previous reports demonstrated that the monomeric peptide RRWQWR does not exhibit a cytotoxic effect against breast cancer cells MDA-MB-231, MDA-MB-468, and MDA-MB-231, as well against other cancer cell lines,^16,19,25,29^ suggesting that the polyvalence of the RRWQWR motif significantly increases the selective cytotoxic effect against human cell lines derived from breast and oral cancer. The mechanism suggested for the antibacterial and anticancer activity of LfcinB involves the initial electrostatic interaction between positive side chains of peptide with the negative charges of the surface cell. The tetrameric peptide contains four copies of RRWQWR sequence, enhancing the number of both kind of amino acids, *i.e.* charged positively (Arg) and hydrophobic (Trp) residues. It has been suggested that the increasing of the positive charge of the peptides make stronger the interaction with the cell surface negative charged molecules.^12–14,32,33^ Also, it has been suggested that LfcinB derived peptides self-assemble, forming polymeric structures, as a requisite for the interaction with the bacterial surface.^13,34^ Then, the Trp residues penetrate into the interfacial layer of the membrane and interact with the lipidic bilayer causing membrane disruption. Furthermore, it has been proposed that Trp induces peptide internalization towards an intracellular target.^22,35–37^

**Fig. 3:**
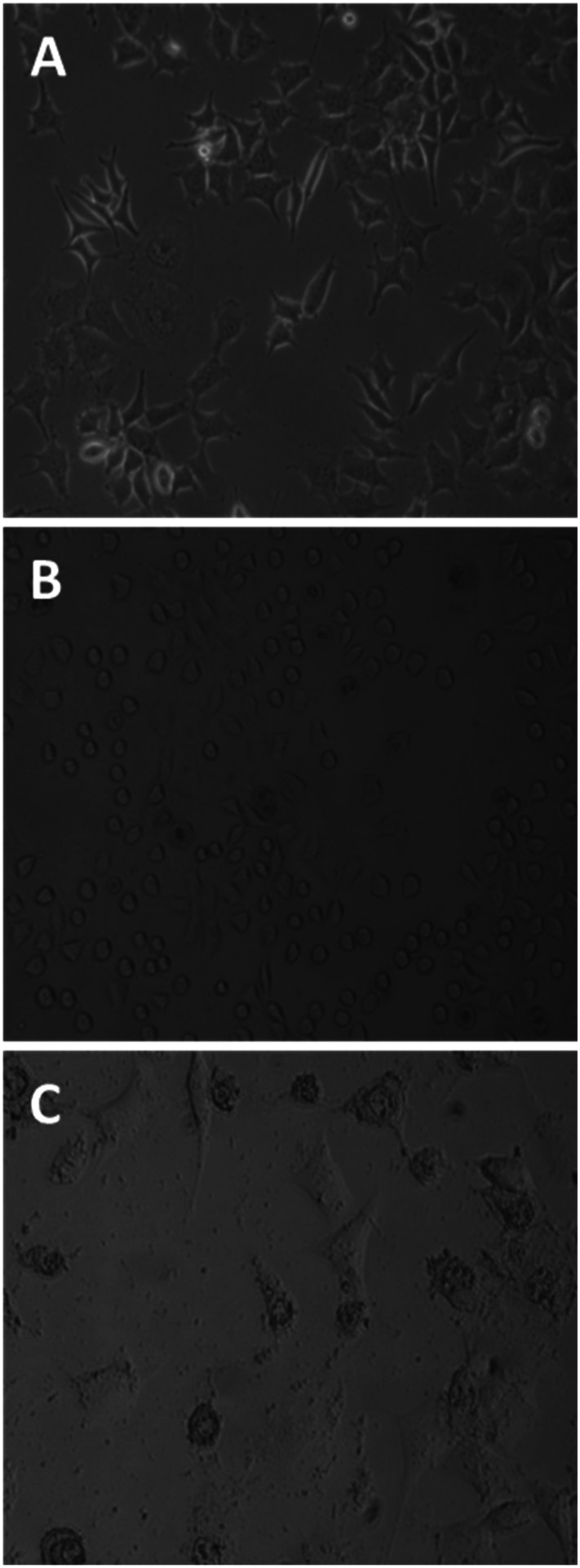
Effect of peptide LfcinB (20–25)_4_ on breast cancer cells MCF-7 (phase-contrast microscopy). Untreated cells (A); MCF-7 cells treated with tetrameric peptide (30 µg mL^−1^; 12.2 µM) for 1 h (B) and 6 h (C).

Untreated MCF-7 cells grown in plastic flasks in a culture medium displayed typical epithelial patterns: a cluster of cells growing in a polygonal shape (Fig. 3A). MCF-7 cells treated for 1 h with the tetrameric peptide showed a marked change in morphology, acquiring rounded forms and shrinkage (Fig. 3B); after 6 h of treatment, MCF-7 cells showed severe damage and cellular lysis (Fig. 3C). These morphologic changes are characteristic of cells undergoing apoptotic processes, suggesting that the cytotoxic effect of the peptide LfcinB (20–25)_4_ is concomitant with apoptotic events.

**Fig. 4:**
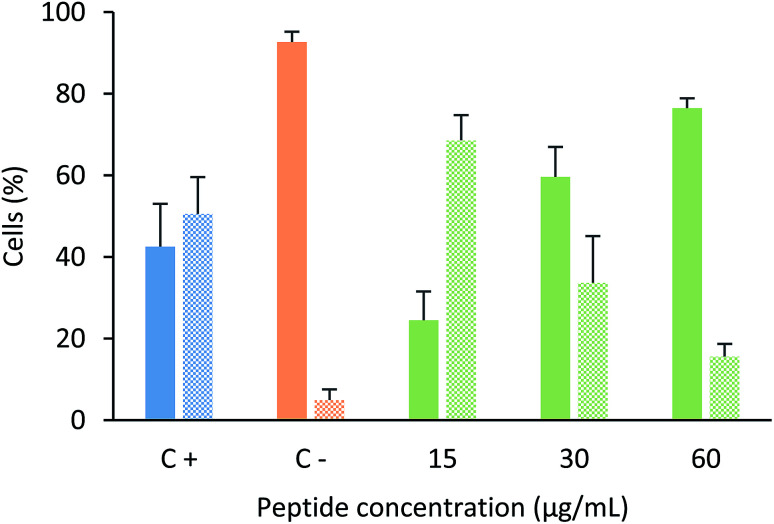
Cytoplasmic membrane integrity assays. MCF-7 cells were treated with tetrameric peptide LfcinB (20–25)_4_ for 6 h in the presence of SYTO9/IP. Plain bars represent cells labelled with SYTO9 fluorochrome (cells with unaffected cytoplasmic membrane). Textured bars represent cells labeled with both SYTO9/PI fluorochromes (cells with affected cytoplasmic membrane). Positive control: cells treated with actinomycin for 24 h (blue); negative control: cells without treatment (orange); cells treated with peptide (green) at 15 µg mL^−1^ (6.6 µM); 30 µg mL^−1^ (12.2 µM) and 60 µg mL^−1^ (24.4 µM). ± SD (*n* = 6).

In order to determine if this tetrameric peptide induces cytoplasmic membrane affectation, MCF-7 cells were treated with the peptide in the presence or absence of SYTO 9/IP fluorochromes (Fig. 4). When MCF-7 cells were treated with the peptide at a concentration of 6.6 µM (15 µg mL^−1^), 69% of cells exhibited an impairment in the cytoplasmic membrane, suggesting that the peptide mainly affects the cytoplasmic membrane and that the peptide's cytotoxic effect could be due to a necrotic process. Furthermore, when cells were treated with peptide at 12.2 or 24.4 µM, the majority of the cells did not exhibit damage in the cytoplasmic membrane, indicating that the cytotoxic effect of the tetrameric peptide against MCF-7 cells can mainly be associated with apoptosis.

These results are in agreement with previous studies that suggest that the cytotoxic effect of LfcinB or peptides derived from LfcinB could be mediated by both apoptosis and/or necrosis.^28^ It has also been shown that this duality in the action mechanism is dependent on the peptide concentration. The anti-tumoral activity of the tetrameric peptide LfcinB (20–25)_4_ in a hamster model was related to both apoptotic and necrotic processes;^28^ the antitumoral activity of animals chronically treated with tetrameric peptide at higher concentrations was mediated mainly by apoptosis.

**Fig. 5:**
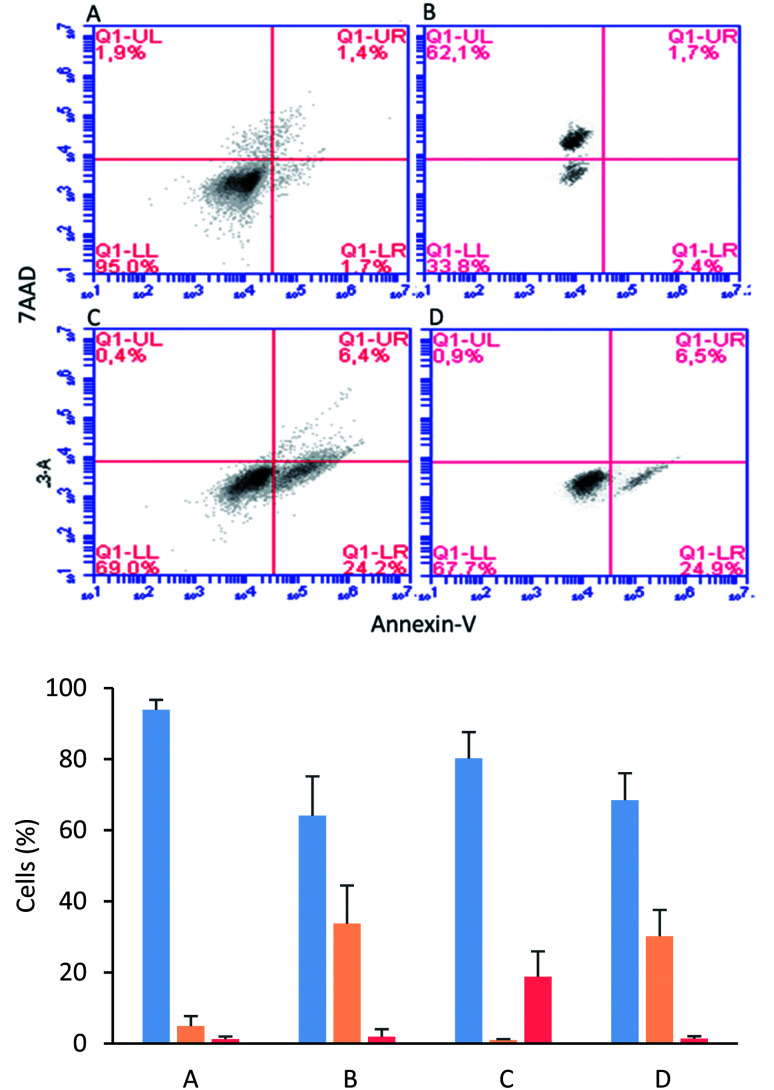
Apoptosis/necrosis assays *via* flow cytometry with Annexin-V and 7AAD. Live cells (blue), apoptotic cells (orange), and necrotic cells (red). Cells in absence of peptide (A), cells treated with actinomycin (B), cells treated with EDTA (C), cells treated with 30 µg mL^−1^ of LfcinB (20–25)_4_ for 1 h (D).

The MCF-7 cells were incubated with tetrameric peptide (12.2 µM) for 1 h, and then the cells were treated with Annexin V/7AAD fluorochromes. The flow cytometry assays showed that 30% of the population corresponded to apoptotic cells similar to the cells treated with actinomycin (apoptosis control; Fig. 5B and D). On the other hand, the necrotic cellular population was less than 2%, suggesting that the cytotoxic effect of the tetrameric peptide involved early events of apoptosis. Similarly, it has been reported that LfcinB exhibited a cytotoxic effect against linfoma B cells, causing DNA fragmentation, chromatin condensation, and nuclear disintegration, suggesting that the cell death was mediated by apoptosis.^38^ In addition, the LfcinB cytotoxic effect against the gastric cancer cell line AGS was selective, concentration-dependent, and mediated by apoptosis.^17^

**Fig. 6:**
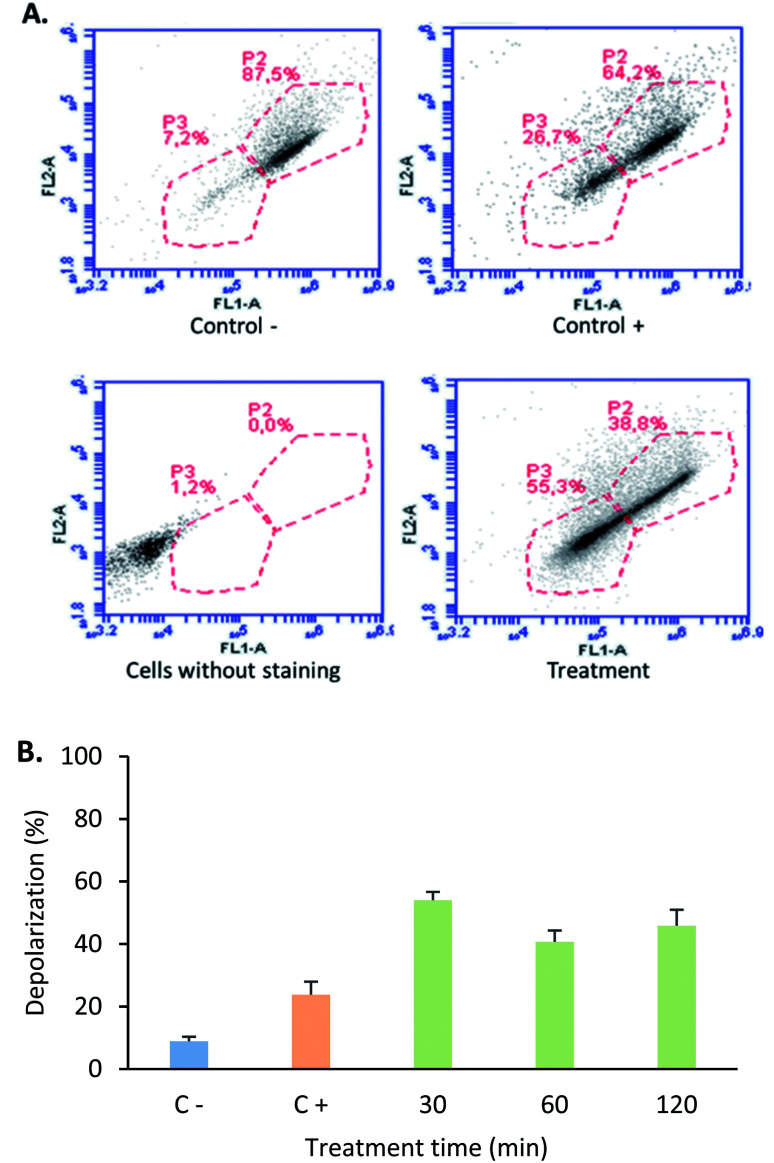
Mitochondrial membrane depolarization assays. (A) Representative images of the flow cytometry of mitochondrial depolarization in MCF-7 cell line. (B) Depolarization percentage of C^−^: cells without treatment (blue), C^+^: MCF-7 cells treated with 10 µg mL^−1^ actinomycin treatment for 24 hours (orange), cells treated with LfcinB (20–25)_4_ peptide (30 µg mL; 12.2 µM) for (green) 30 min, 60 min and 120 min.

The mitochondria play a critical role in cell death regulation, and their membrane permeabilization occurs in the initial phase of the intrinsic apoptosis pathway, releasing proteins and calcium into the cytoplasm. With the aim of establishing if the tetrameric peptide's cytotoxic effect against MCF-7 cells involves mitochondrial membrane depolarization, the cells were treated with peptide (12.2 µM) at 0.5, 1, and 2 h, and then were stained with JC1 fluorochrome. For MCF-7 cells treated with tetrameric peptide at 0.5, 1, and 2 h, the cellular population percentage with depolarized mitochondria was 54%, 41%, and 46%, respectively, suggesting that the tetrameric peptide induces depolarization in the mitochondrial membrane of these breast cancer cells. The cellular population with depolarized mitochondrial membrane induced by the tetrameric peptide was higher than those treated with actinomycin (24%) (Fig. 6).

Mitochondrial membrane depolarization induced by tetrameric peptide is in agreement with the results observed in apoptosis/necrosis assays, indicating that the mechanism associated with the cytotoxic effect of the tetrameric peptide against breast cancer cells MCF-7 could be mediated by the intrinsic apoptotic pathway.

In accordance with our results, some peptides containing the minimal motif induced apoptosis in leukemia cell line Jurka T; the mechanism proposed for the cytotoxic effect of the peptide FKCRRWQWRM against Jurkat T cells involves peptide internalization, cytochrome C, and reactive oxygen species (ROS) liberation.^19^ In addition, the internalization of LcinB (20–25) in these cells was associated with cathepsin B and caspase-dependent DNA fragmentation.^25^ LfcinB exhibited a dose- and time-dependent selective cytotoxic effect against breast cancer cells lines MCF-7, T-47D, and MDA-MB-435.^19^ Furthermore, LFcinB exhibited cytotoxicity against Jurkat 1, THP-1, and MDA-MB-435 cells mediated by apoptosis. In Jurka T cells, LfcinB induced intracellular production of ROS and activation of Ca^2+^/Mg^2+^-dependent endonucleases.^19,20,39^ LfcinB induces mitochondrial-dependent apoptosis in leukemia, lymphoma, and breast cancer cell lines.^19,38–41^

**Fig. 7:**
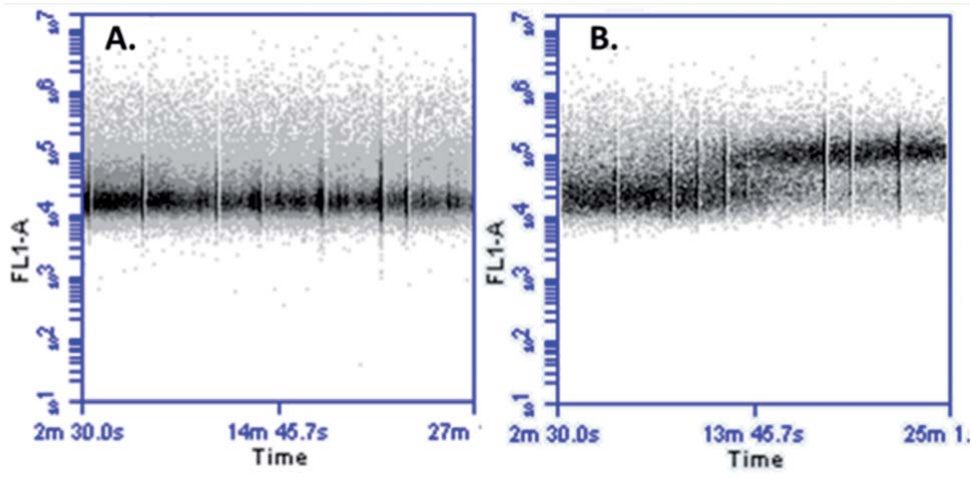
Cytoplasmatic calcium measurement in MCF-7 cells treated with LfcinB (20–25)_4_. (A) Negative control untreated cells and (B) cells treated with LfcinB (20–25)_4_; the peptide (30 µg mL^−1^) was injected at 5 min.

In cellular death mediated by apoptosis, cellular calcium overload, mitochondria take up cytosolic calcium, which in turn induces the opening of the permeability pores and disrupts the mitochondrial membrane potential. Mitochondrial calcium overload is one of the pro-apoptotic ways to induce the swelling of mitochondria and membrane rupture, releasing mitochondrial apoptotic factors into the cytoplasm.^42,43^ It has been suggested that an increase in cytoplasmic calcium might be associated with apoptotic signalling. With the aim of determining if the cytotoxic effect of peptide LfcinB (20–25)_4_ against MCF-7 cells affects the calcium intracellular concentration, cells were loaded with fluorescent calcium indicator and then treated with the peptide. When the cells were exposed to the tetrameric peptide for 5 min, an increase of fluorescence was observed, which was maintained for 23 min, suggesting that the peptide induces increasing cytoplasmic calcium concentration in MCF-7 cells (Fig. 7). The change of cytoplasmic calcium concentration caused by the tetrameric peptide is in agreement with results obtained above, suggesting that the tetrameric peptide exhibited a cytotoxic effect against MCF-7 cells through the apoptosis route.

## Material and methods

### Reagents and materials

MCF-7 and CRL-3271 cells were obtained from ATCC (Manassas, VA, USA), fetal bovine serum (FBS) was obtained of Gibco. MitoProbe™ JC-1 Assay Kit (Termofisher; M34152), Calcium Assay Kit (BD Biosciences; 640176), 7AAD and Annexin V (BD Biosciences; 559763) *N*,*N*-diisopropylethylamine (DIPEA), triisopropylsilane (TIPS), 1,2-ethanedithiol (EDT), 4-methylpiperidine, pyridine, and ninhydrin were obtained from Sigma-Aldrich (St. Louis, MO, USA). Rink amide resin, Fmoc-amino acids, 6-chloro-1-hydroxy-benzotriazole (6-Cl–HOBt), and *N*,*N*-dicyclohexylcarbodiimide (DCC) were purchased from AAPPTec (Louisville, KY, USA). Methanol, diethyl ether, *N*,*N*-dimethylformamide (DMF), absolute ethanol, dichloromethane (DCM), acetonitrile (ACN), isopropylalcohol (IPA), and trifluoroacetic acid (TFA) were obtained from Honeywell-Burdick & Jackson (Muskegon, MI, USA). All reagents were used without further purification.

### Solid phase peptide synthesis

In a previous paper we reported the synthesis of LfcinB (20–25)_4_ peptide by oxidation of the Precursor Dimeric Peptide (PDP): (RRWQWR)_2_-K-Ahx-C. PDP was synthesized using the MAPs (multiple antigen peptides) methodology. The PDP peptide was obtained with high chromatographic purity, and the purified peptide had the expected monoisotopic mass.^16^ The methodology used by Vargas *et al.*^16^ is describe as follows: tetrameric peptide was obtained for the oxidation of precursor dimeric peptide (PDP): (RRWQWR)_2_KAhxC according to the methodology described by Vargas *et al.*^16^ PDP was synthesized using the manual SPPS-Fmoc/*t*Bu methodology. For this, Fmoc-Lys(Fmoc)-OH was used in order to simultaneously build two chains containing the motif RRWQWR. Aminoexanoic acid was used as a spacer to facilitate the synthesis, and cysteine amino acid was used to form the disulfide bond between two PDP, which leads to tetrameric peptide formation. Rink amide resin (0.46 meq. g^−1^) was used as a solid support. (i) Fmoc group removal was carried out through treatment with 20% 4-methylpiperidine in DMF. (ii) For the coupling reaction, Fmoc-amino acids (0.21 mmol) were pre-activated with DCC/6-Cl–HOBt (0.20/0.21 mmol) in DMF at RT. (iii) Side-chain deprotection reactions and peptide separation from the resin were carried out with a cleavage cocktail containing TFA/water/TIPS/EDT (93/2/2.5/2.5 v/v/v). (iv) Crude PDP was precipitated by treatment with cool ethyl ether, dried at RT, and analyzed using RP-HPLC analytical chromatography. PDP was purified using solid-phase extraction columns (SUPELCO LC-18 with 2.0 g resin).^44^ SPE columns were activated prior to use with 30 mL acetonitrile (containing 0.1% TFA) and equilibrated with 30 mL water (containing 0.1% TFA). Crude PDP was passed through the column, and a gradient was used for their elution. Collected fractions were analyzed using RP-HPLC.^16^ Fractions that contained the pure product were lyophilized. PDP oxidation was carried out by dissolving the PDP (chromatographic purity 95%) in water (containing 0.1% TFA), and then the pH was adjusted to 8.0 using NH_4_HCO_3_. The solution was gently stirred at room temperature, and atmospheric oxygen was passed through the solution until complete oxidation of PDP. The oxidation reaction was monitored *via* RP-HPLC, and the tetrameric peptide was purified using SPE chromatography, as described above.

### Cytotoxic assays

Cytotoxicity assays were performed as previously described by Vargas *et al.*^16^ Briefly, MCF-7 or CRL-3271 cells (8 × 10^3^ cells per well) were seeded in 96-well plates. After cell adherence, the culture medium was removed and the cells were treated with tetrameric peptide (200 to 6.25 µg mL^−1^) for 2 or 24 h at 37 °C. Cell viability was determined using the MTT assay; for this, 10 µL of MTT solution (5 mg mL^−1^) was added to each well and the plates were incubated for 4 h at 37 °C. Formazan crystals were dissolved in DMSO (100 µL), and absorbance (570 nm) was recorded on a Bio-Rad 680 microplate reader. Negative control: cells incubated with medium, (*n* = 3).

### Cytoplasmic membrane integrity assays

The MCF-7 cells (6 × 10^5^ cells per well) were seeded in 96-well plates. After cell adherence, the culture medium was removed and the cells were treated with tetrameric peptide (15, 30 or 60 µg mL^−1^) for 6 h at 37 °C. After the cells were harvested with trypsin and centrifuged at 2500 rpm for 5 min and the pellet was washed with PBS. After the cells were labeled with 30 µL of a solution of the commercial kit LIVE/DEAD® FungaLight™ (0.5 µL of SYTO9 and 0.5 µL of Iodide of Propidium (IP) with 99 µL of PBS) according to the manufacturer's protocol. Then the cells were incubated at RT for 20 min and then centrifuged, and the pellet was resuspended in 100 µL of PBS and analyzed *via* flow cytometry in BD Accuri C6 equipment. Negative control: cells incubated with medium; positive control: cells treated with actinomycin 10 µg mL^−1^ for 24 h.

### Apoptosis/necrosis assays

The MCF-7 cells (6 × 10^5^ cells per well) were seeded in 96-well plates. After cell adherence, the culture medium was removed and the cells were treated with tetrameric peptide (30 µg mL^−1^) for 1 hour at 37 °C. After the cells were harvested with trypsin, centrifuged at 1800 rpm, washed with PBS and suspended. The cells were labeled with Annexin V/7AAD using the Dead Cell Apoptosis Kit for flow cytometry (Thermo Fisher Scientific) (10 mM Hepes pH 7.4, 10 mM NaCl and 2.5 mM CaCl_2_, 1 µL of 7AAD fluorochrome and 1 µL of Annexin V) according to the manufacturer's protocol. The cells with the fluorochromes were incubated at 37 °C for 15 min and resuspended in 80 µL of staining buffer without fluorochromes and analyzed by flow cytometry. Phase contrast photomicrographs of the unlabelled treated cells were taken (Leica Leits). Control for necrosis: cells were treated with 15 mM of EDTA for 20 min. Control for apoptosis: cells treated with 10 µM of actinomycin at for 24 hours. Negative control: cells without treatment.

### Mitochondrial membrane depolarization assays

The MCF-7 cells (6 × 10^5^ cells per well) were seeded in 96-well plates. After cell adherence, the culture medium was removed and the cells were treated with tetrameric peptide (30 µg mL^−1^) for 0.5, 1 and 2 h at 37 °C. Then the cells were trypsinized and harvested by centrifugation at 400*g* × 5 min. Then the cells were labeled by adding 100 µL of JC1 “working solution” (1 : 100 JC1 in DMSO : buffer assay 1×) and incubating at 37 °C for 20 min (MitoProbe™ JC-1 assay kit M34152; Thermo Fisher Scientific) according to the manufacturer's protocol. Then the cells were washed twice with “buffer assay 1×” and finally they were suspended in 100 µL of buffer assay 1×. Negative control: cells without treatment. Positive control: cells treated with actinomycin 10 µg mL^−1^ for 24 hours.

### Determination of intracellular calcium release

MCF-7 cells (6 × 10^5^ cells per well) were seeded in 96-well plates. After cell adherence, the culture medium was removed and the cells were treated with tetrameric peptide (30 µg mL^−1^) for 1 h at 37 °C. The cells were labeled with 10 µL of “calcium indicator” and 10 mL of “signal enhancer 1×”, (Calcium Assay Kit 640176, DB Biosciences) according to the manufacturer's protocol. Negative control: cells treated with incomplete medium.

## Conclusions

The peptide LfcinB (20–25)_4_ exhibited a selective cytotoxic effect against the MCF-7 cell line. This cytotoxic effect is concentration-dependent and is maintained for 24 h. In a similar way, the tetrameric peptide also exhibited a selective cytotoxic effect against breast cancer cells HTB-132 and HTB-26, as well against the oral cancer cells CAL-27 and SCC15. According to this, the tetrameric peptide is an anticancer molecule with a broad-spectrum cytotoxic effect in breast and oral cancer cell lines. The tetrameric peptide exhibited higher cytotoxic effect against breast cancer cells than peptide LfcinB (20–25), suggesting that the polyvalence of the RRWQWR sequence significantly increases the anticancer activity. The tetrameric peptide induces severe cellular damage associated with mitochondrial membrane depolarization, increase in intracellular calcium, and morphologic changes characteristic of cells suffering apoptosis. Thus the design of polyvalent peptides containing the RRWQWR sequence increases antibacterial and anticancer activity and is a viable and novel strategy that allows obtaining selective peptides with a significant cytotoxic effect against human breast cancer cell lines. The tetrameric peptide LfcinB (20–25)_4_ could be considered to be a promising molecule for future studies in order to establish its potential use against breast cancer.

## Conflicts of interest

There are no conflicts to declare.

## Supplementary Material
